# Proteomics Impact on Cell Biology to Resolve Cell Structure and Function

**DOI:** 10.1016/j.mcpro.2024.100758

**Published:** 2024-04-02

**Authors:** John J.M. Bergeron

**Affiliations:** Department of Medicine, McGill University Hospital Research Institute, Montreal, Quebec, Canada

**Keywords:** molecular/virtual microscope, proteomics, cryo-electron tomography, cryo-EM, cell structure

## Abstract

The acceleration of advances in proteomics has enabled integration with imaging at the EM and light microscopy levels, cryo-EM of protein structures, and artificial intelligence with proteins comprehensively and accurately resolved for cell structures at nanometer to subnanometer resolution. Proteomics continues to outpace experimentally based structural imaging, but their ultimate integration is a path toward the goal of a compendium of all proteins to understand mechanistically cell structure and function.

## Highlights

• The nuclear pore complex.
• Motile cilia.
• The centrosome.
• Mitotic chromosomes.
• The ribosome.

## Graphical Abstract

**Figure:**
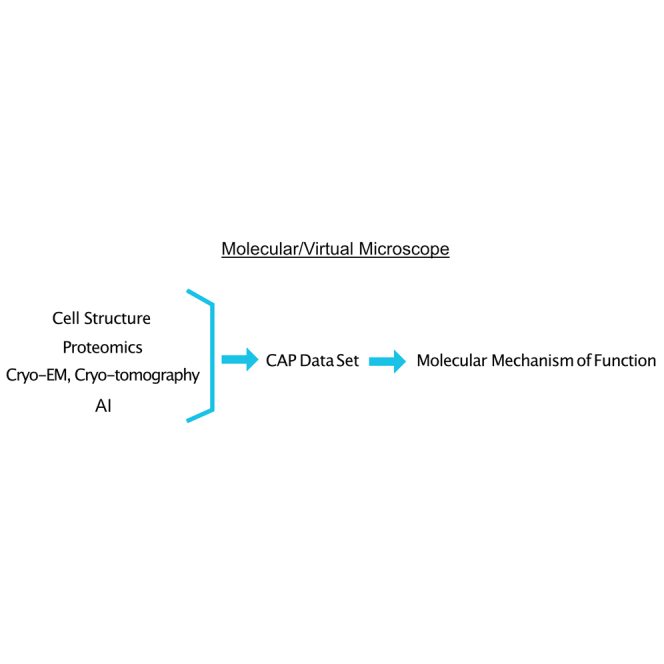

## In Brief

The proteomics foundation to resolve cell structure and function as the basis for the integration with marked advances in EM imaging is available to the cell biology community.

The near-complete characterization of proteins in cell structures has been propelled by quantitative proteomics linked to quantitative imaging of the proteins at the EM level supplemented by artificial intelligence (AI). Today, the technical hurdles are being overcome (1) because of pioneering efforts over decades to establish a pipeline as indicated in the graphical abstract.

For Cell Biology, progress from the compendium of cell structures as visualized by EM from pioneers of the last century (2) to progress in proteomics to detailed mechanisms of function through a comprehensive visualization of proteins on the same cell structures is noteworthy. Selected examples include the nuclear pore complex, motile cilia, the centrosome, mitotic chromosomes, and the ribosome. The studies have approached and, in some cases, attained the goal of a near-comprehensive, accurate, and permanent compendium of the proteins in cell structures that have also enabled their mechanism of function to be resolved.

## The CAP Goal

It was Sydney Brenner who proposed the CAP (Comprehensive, Accurate, and Permanent) criteria indicated in the graphical abstract for large-scale data (3). CAP criteria had been demonstrated by Brenner’s mapping of the synaptic connections (the connectome) by serial section EM of the 302 neurons in the nematode *Caenorhabditis elegans* in 1986 (4). The task took 14 years with over 10,000 electron microscopic thin serial sections imaged and mapped manually. This work remains the basis for the yet unsolved connectome of the vertebrate brain (5).

**Fig. 1:**
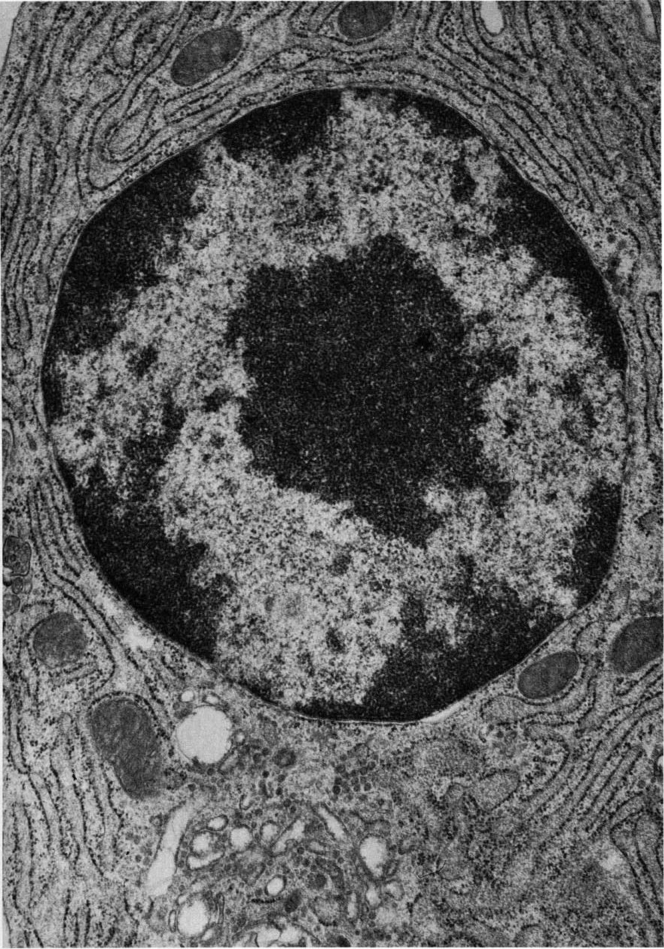
**A plasma cell nucleus is encompassed by the nuclear envelope in the cytoplasm of the cell.** Reproduced from Fawcett 1981 (Fig. 114) (2).

**Fig. 2:**
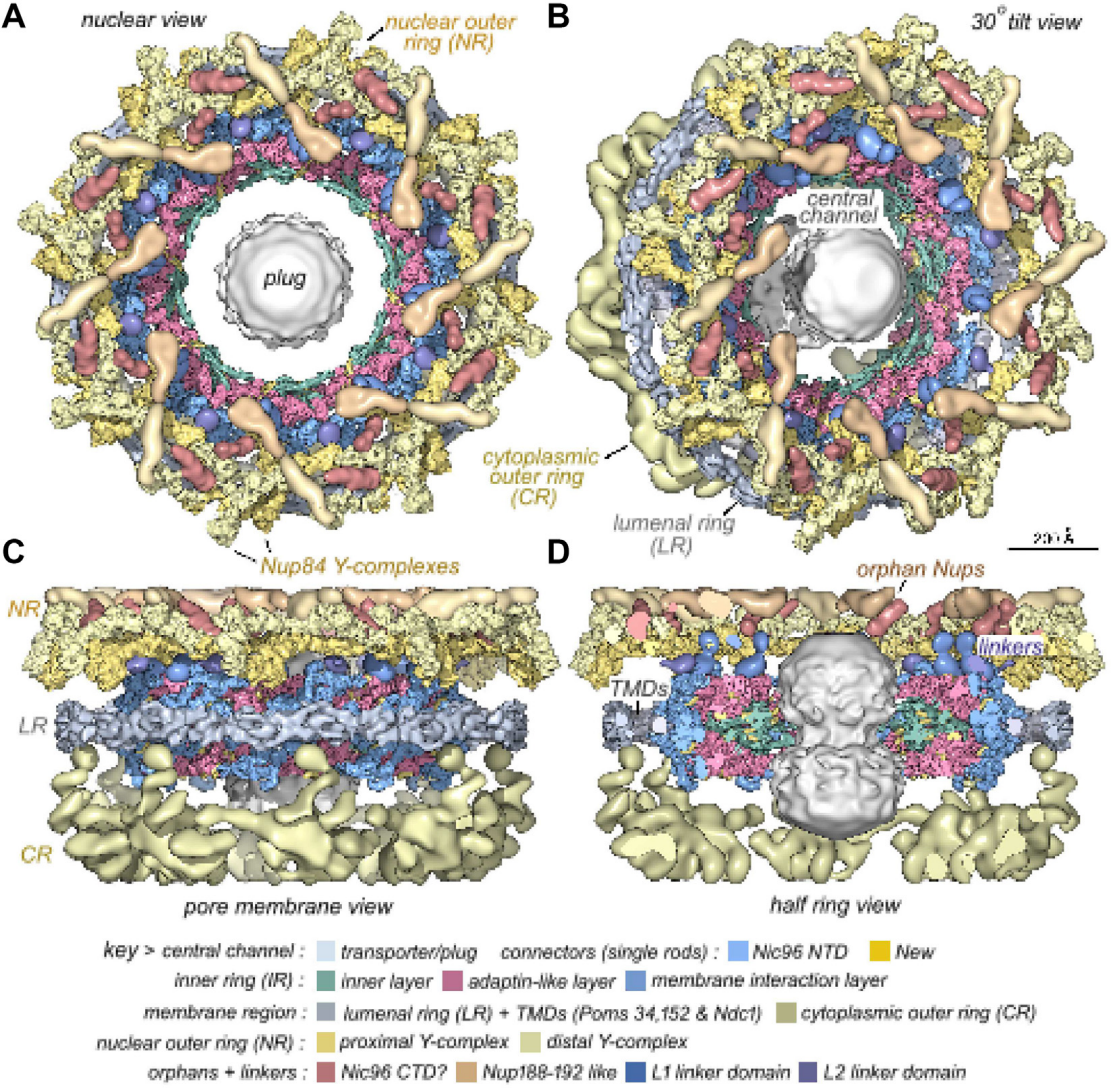
**3D structure of the yeast nuclear porecomplex.** This image reveals the nuclear view (*A*), the tilted nuclear view (*B*), the pore membrane view (*C*), and the half-ring view (*D*). Adapted from Akey *et al.*, 2023 (6).

Today, the CAP goal appears to have been reached for the nuclear pore complex of eukaryotes. The early EM description of a nucleus (Fig. 1) has progressed to a near-complete understanding of the protein makeup and an increasingly deep understanding of the mechanism of function of the nuclear pore harboring the entry and exit points of the nucleus (Fig. 2). The image of Figure 1 may be contrasted with the molecular microscope resolution of the nuclear pore complex today in Figure 2.

## The Nuclear Pore Complex

The elucidation of the nuclear pore complex has emerged from more than 30 years of progress.

The strategy of a pipeline consisting of sample preparation, protein characterization, stoichiometry, protein partners, and complete structural elucidation including the use of AI complemented with *in situ* imaging deduced a model for the mechanism of function. Taken together, the work from yeast to humans has extended considerably how the challenges previously raised for integrating proteomics data for cell biology (6, 7) are available to cell biologists (1).

For yeast, it is at the level of sample preparation on which a pyramid of discovery rests. In 1993, Rout and Blobel (8) isolated the nuclear pore complex from the model organism, budding yeast. EM confirmed an eightfold symmetry of the isolated yeast nuclear pore structure that has stood the test of time. Most of the 80 proteins they uncovered that coisolated with the nuclear pore complexes were unknown at the time.

Progress in the structural and functional characterization of the nuclear pore complex came from an integrative combination of multiple orthogonal approaches as a conceptual “molecular microscope” (9). Mass spectrometry enabled the characterization of proteins in the isolated nuclear pore complex (10). Of the total of 174 proteins identified, 30 were concluded to be genuine constituents of the nuclear pore complex and confirmed as nucleoporins.

Stoichiometry was assessed by quantitative immunoblotting of the tagged proteins to estimate three categories of nucleoporins: at 8, 16, or 32 copies per nuclear pore complex. The characterization of protein–protein interactions in the nuclear pore complex and further assessment of stoichiometry by mass spectrometry followed for yeast (11, 12) and independently by Beck *et al.* (13, 14)for human nuclear pore complexes.

Exhaustive optimization of affinity purification protocols enabled complexes in yeast to be characterized that also could be assessed for imaging (15, 16, 17, 18, 19). With cryogenic samples, microscopy defined the spatial location of each protein of the nuclear pore complex (12, 20). Protein crosslinking with disuccinimidyl suberate and separation by SDS-PAGE resolved the proximal residues of interacting proteins or neighboring proteins based on tandem mass spectrometry of tryptic digests of cross-linked proteins separated by SDS-PAGE (20). This provided “NMR-like” distance restraints between nucleoporins with 3077 unique cross-linked pairs of residues that aided the calculation of the unambiguous spatial molecular architecture of the complex. Charge detection mass spectrometry was used to determine the total mass of the tag-isolated nuclear pore complex. A mass of 52 MDa was determined for the isolated nuclear pore complex, which increased to 87 MDa when membrane proteins, cargo proteins, and transport factors were incorporated.

The detailed cross-linking studies in yeast complemented and extended prior independent studies using proximity-dependent biotin identification (BioID) in human cells that did not require prior isolation of nuclear pore complexes (21). More complexes were characterized by BioID than observed at that time for cross-linking mass spectrometry of isolated human nuclear pore complexes with 17 confidently assigned for the latter (22). However, the labeling radius for proteins able to be characterized by mass spectrometry after BioID pull down was measured as ca. 10 nm rather than the higher resolution ca. 2 nm by crosslinking.

Yeast studies were further extended by using cryogenic samples to isolate nuclear pore complexes and visualization by electron cryotomography. The integration of images from different tilt angles of the cryogenic nuclear pore complexes in the EM generated a 3D tomogram (23). Using the accurate localization of all cross-linked residues of the yeast nuclear pore complex as determined by mass spectrometry, all 552 proteins in the nuclear pore complex were resolved (24).

Protein structures solved by cryo-EM were supplemented by AI (25). AlphaFold2 assigned missing structures with the images coherent with the stoichiometry from quantitative mass spectrometry to define the complete structure, accurately, and comprehensively. This included rare yeast nuclear pore complexes with double outer rings (25). Double outer rings are characteristic of vertebrate nuclear pore complexes (22).

The detailed structure (Fig. 2) in yeast (25) confirms the architecture of the earlier observed eightfold symmetrical structures of the nuclear pore complex (8). This now includes how the eight spokes link the nuclear pore complex to the nuclear envelope. The spokes and associated inner and outer rings are linked to the central transporter filled with nucleoporin FG (phenylalanine, glycine) repeat proteins that are interspersed with abundant transport factors. The entire nuclear pore complex reveals an overall structure reminiscent of how a suspension bridge enables high-volume transport while resisting stress (24).

## A Mechanism of Function of the Nuclear Pore Complex

**Fig. 3:**
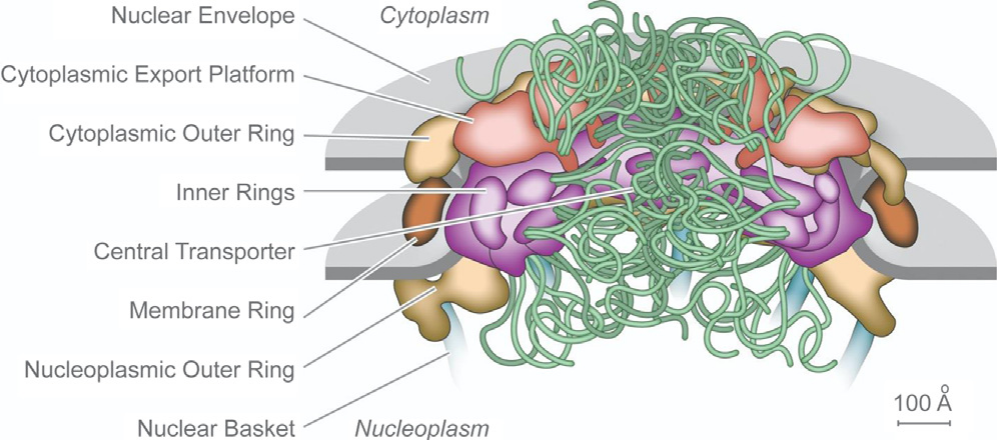
**Schematic of the nuclear pore complex of budding yeast showing the central transporter filled with the intrinsically disordered FG nucleoporins that select nuclear transport receptors with associated cargo for import and export across the nuclear pore.** Reproduced from Cowburn and Rout, 2023 (23). FG, phenylalanine, glycine.

For Cell Biology, one goal is to solve the molecular mechanism of function. It is through the molecular microscope (graphical abstract) that this may have been achieved for the nuclear pore complex. The yeast nuclear pore complex is about 100 nm in diameter with a center of about 50 nm diameter representing the central transporter (Fig. 3) (26). The central transporter prevents the passage of nonspecific macromolecules with metabolites and ions small enough to passively diffuse through the nuclear pore.

An early model deduced from the proteomics-based molecular microscope proposed a “virtual gate” to explain how the protein constituents of the central transporter regulate access to or exit from the nuclear interior (10). The virtual gate mechanism is now understood as an entropic/enthalpic barrier of the central transporter through the action of the FG-repeat nucleoporins. Although intrinsically disordered proteins, they are now understood to be in an extended conformation (27).

The central transporter is packed with nuclear transport receptors and their protein or RNA cargo as deduced by quantitative mass spectrometry using BioID by the Beck group (28). Together with the FG-repeat nucleoporins, they form a barrier to prevent cargo not associated with cargo carriers from entering or exiting through the nuclear pore complex. Transport is enabled by the offset of entropy and enthalpy. The entropy component is derived from the restriction of cargo carriers within the small channel, further constrained by the dynamic motion of intrinsically disordered FG-repeat nucleoporins, randomly batting out unsolicited cargo. Enthalpy is derived from nuclear transport receptors that use the enthalpy associated with binding to FG domains to carry their cargo across the channel by hopping between FG repeats within and between nucleoporins. The FG proteins interact with several sites on transport receptors with the latter hopping on the FG-repeat proteins with an on/off time scale of microseconds (26).

The Beck group has studied extensively the vertebrate nuclear pore complex. It has a similar inner ring but differs from that in yeast by having two outer rings while retaining similar architectural principles to the nuclear pore complex of yeast (22). The proteomics of isolated nuclear pore complexes from mammalian sources provided a parts list that was quantitative (29). This could now be extended to the use of integrated datasets from proteomics and EM cryotomography to elucidate the human nuclear pore complex (22). This study also utilized cross-linking mass spectrometry to characterize protein complexes followed by their visualization through cryo-electron tomography of isolated nuclear envelopes. It is the matching of protein structures with complexes visualized based on the cross-linking mass spectrometry studies (30) that resulted in the resolved nuclear pore complex (22). Additional phosphoproteomics studies (22) revealed insight into how the nuclear pore complex is assembled.

Further studies using the integration of data from cryo-electron tomography and mass spectrometry provided a more comprehensive elucidation of the human nuclear pore complex (31). The study gave insight as to how different concentric rings of the nuclear pore complex can be assembled from the same building blocks as well as documenting their molecular interactions. As well, insight into the relevance of protein phosphorylation and the onset of mitosis was shown.

The integrated data were extended further for the human nuclear pore complex now with the additional application of AI-based structure determinations from AlphaFold2 and Rose TTA fold (32). In this way, the structure was resolved for most proteins of the cytoplasmic ring as well as the nuclear and inner rings to near-atomic resolution. Insight into the mechanism of nuclear pore transport by the FG domain proteins, the anchoring mechanism of the nucleoporin scaffold proteins to the membrane of the nuclear envelope, and the membrane conformation around the pore was provided. Remarkably, conformational differences in the nuclear pore complex were resolved for nuclear pore constriction and widening (33).

The key role of proteomics has led to the elucidation of the structure and function of the nuclear pore complex through the molecular microscope (9) that has satisfied the Brenner criteria of the CAP principle for the yeast nuclear pore complex and near completion of the human nuclear pore complexes. The independent studies of yeast (Fig. 2) and human nuclear pore complexes (32) revealed the major differences between mammalian and yeast nuclear pore complexes while sharing a common mechanism of transport into and out the nucleus. It is the application of an integrated strategy of data from proteomics and molecular imaging now with the advance of AI that has propelled the field.

## Motile Cilia

**Fig. 4:**
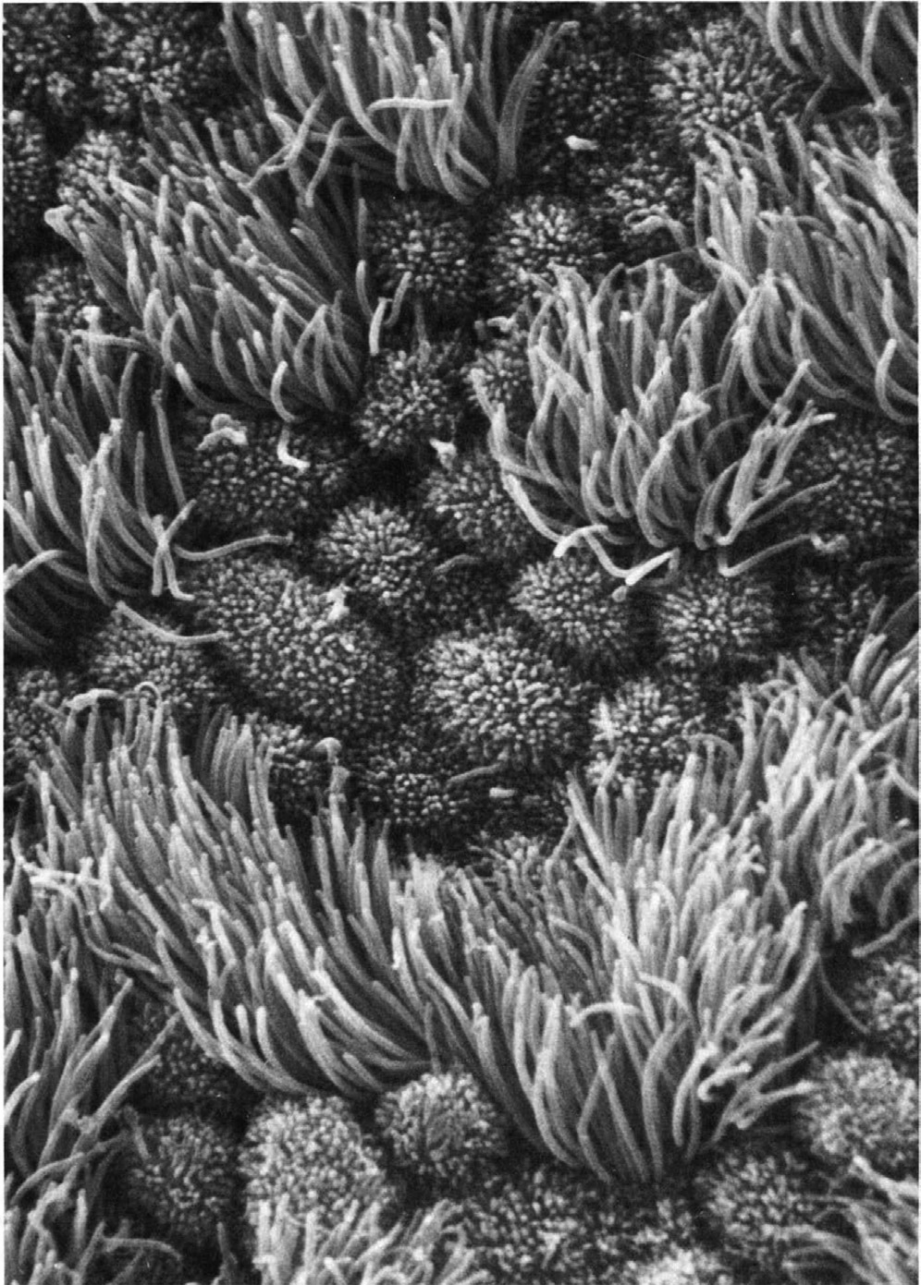
**Scanning micrograph of the epithelium of the human oviduct, at midcycle showing ciliated cells next to nonciliated cells with short microvilli.** Reproduced from Fawcett 1981 (Fig. 316) (2).

Progress in attaining the CAP principle has been made by studies on motile cilia. Motile cilia (Fig. 4) are essential structures in oviducts, respiratory epithelia, brain ventricles, and sperm flagella. As for the nuclear pore, model organisms have been key and have been complemented by studies on human motile cilia, including human sperm flagella.

**Fig. 5:**
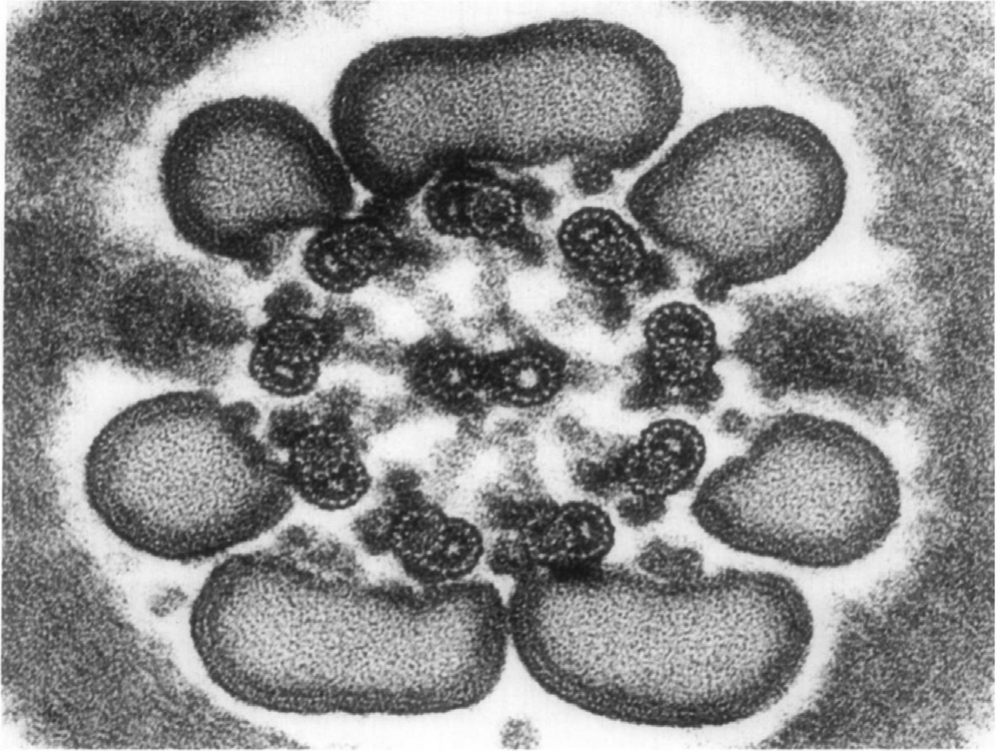
**EM of the axoneme and associated fibers in the principal piece of a spermatozoan flagellum showing the protofibrils in the walls of the two central singlet microtubules and of the surrounding nine doublet microtubules.** The cortex and medulla of the outer fibers are also clearly differentiated. Reproduced from Fawcett 1981 (Fig. 336) (2).

The motility of cilia and flagella is due to the microtubule-based structure known as the axoneme. Progress in the resolution of the protein makeup and visualization at nanometer resolution in motile cilia has also benefitted from model organisms. These include *Chlamydomonas rheinhardtii*, which has biflagellate cilia (34) and conserves the two central singlet microtubules surrounded by nine associated doublet microtubules also for sperm flagella as visualized by EM from the pioneers in cell biology (Fig. 5). From the proteomics of the central apparatus of the singlet microtubules and associated proteins, cryo-EM of Chlamydomonas central apparatus followed (35). The high-resolution study revealed conservation among species and insight into the molecular regulatory mechanism for ciliary beating. For the doublet microtubules, a comprehensive proteomics characterization of the ciliate *Tetrahymena* thermophila using crosslinking of cilia *in situ* identified proteins of the microtubule inner proteins of the inner wall of the doublet microtubules (36). A further study with crosslinking-based proteomics, BioID, pull-down assays, cryo-EM, cryo-electron tomography, and AI-based AlphaFold2 solved a model for the nexin–dynein regulatory complex (37).

**Fig. 6:**
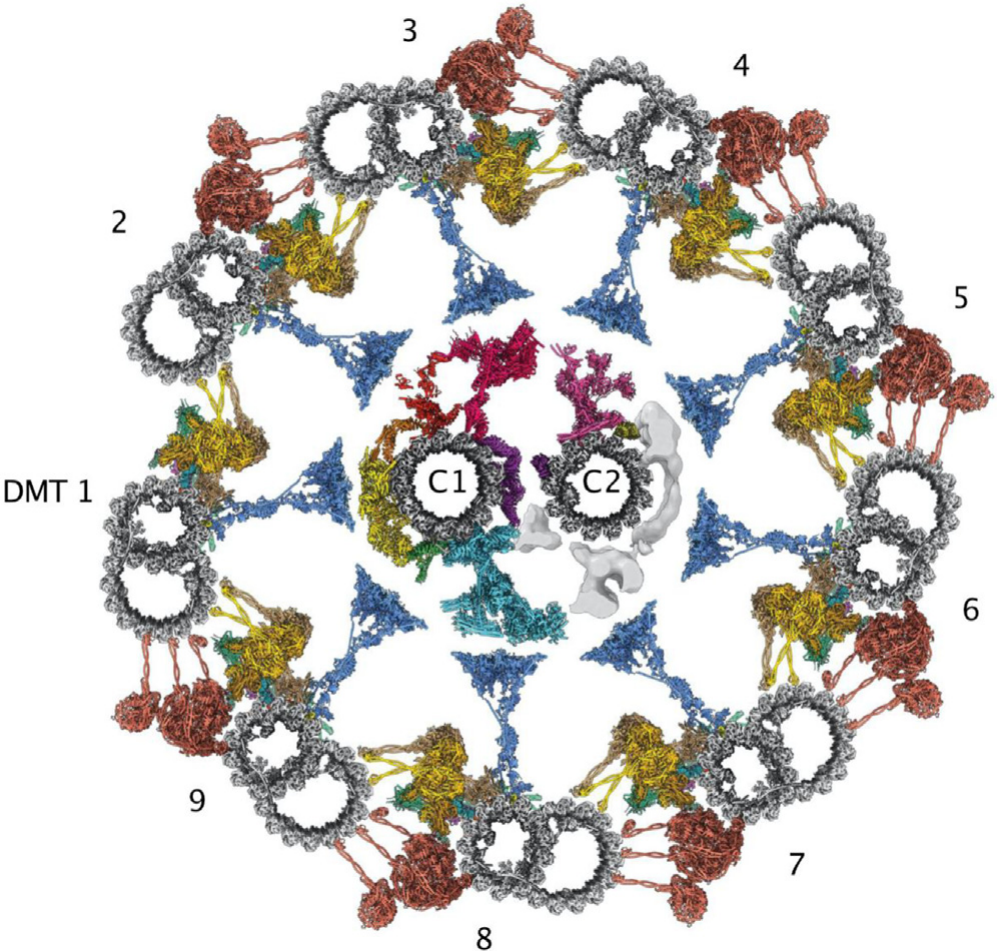
**Cross-section of the axoneme from flagella of *Chlamydomonas reinhardtii* from**Fig. 1*B***of Walton *et al.*** (38)**.** The two central singlet microtubules (C1, C2) surrounded by the nine associated doublet microtubules (DMTs) are indicated. A comparison with the image of sperm flagella (Fig. 5) reveals the noteworthy advance with detailed protein structures extending the basic features of the EM imaging of the axoneme of the last century.

The achievement of CAP criteria has enabled insight into the motile cilia of flagella of *C. rheinhardtii* and the cilia of human respiratory epithelium by Walton *et al.* (38). The molecular mechanisms for regulating ciliary motility have been deduced with a molecular explanation for human patients suffering from primary ciliary dyskinesia. The isolation of axonemes followed by cryo-EM and integration with cryo-electron tomography data and AI has visualized the 9 + 2 structure of the axoneme of *C. rheinhardtii* at near-atomic resolution (Fig. 6). This was extended to human cilia including those from patients with mutations leading to ciliary dyskinesia. A regulation of ciliary beating with a proximal to distal waveform was proposed with a mechanical signal *via* the dynein regulatory complex (38).

From these model organisms that extend to humans and human patients, a near-atomic resolution of the doublet microtubule of the motile cilia also revealed the filamentous microtubule inner proteins to understand how ciliary waveforms are regulated during ciliary beating to propel cell and fluid movement (38, 39, 40).

## The Centrosome

The centrosome is the major microtubule-organizing center in animal cells (41, 42). The structure has a mother-and-daughter centriole that establishes the axoneme for motile flagella as well as nonmotile cilia. During the cell cycle, mother and daughter centrioles are duplicated and segregated to ensure a mother–daughter pair in each new cell after mitosis. A cilium is generated in G1 or G0 *via* the mother centriole’s distal appendage proteins that are absent from the associated daughter centriole. The mother centriole distal appendages are associated with the apical surface of polarized epithelial cells. The seeding and assembly of the axoneme for nonmotile cilia formation are generated and supported by the base mother centriole in association with the daughter centriole. The mother centriole is known as the basal body of the cilium. Fibroblasts use a vesicular transport mechanism of mother centriole association *via* the distal appendage proteins to target the cell surface with membrane fusion and cilia formation from the basal body.

A recent proteomics compendium of proteins of the centrosome has applied a pull-down variation (43) of BioID (44) that has been used extensively for proteomics. Named CAPture (centrosome affinity capture), the method is directed at centrosome isolation from cell lysates (43). A synthetic peptide of 33 amino acids was selected from the protein variable flagellar protein 3, also named CCDC61, coupled to biotin. After incubation with lysates, magnetic beads coupled to streptavidin were used to isolate centrosomes. Protein characterization by LC–MS–MS of tryptic digests was followed with tandem mass tag labeling quantification. The comprehensive results extended past proteomics studies of centrosomes.

Insight into the mechanism of assembly of the proteins of subdistal appendages onto daughter centrioles to become mother centrioles was also demonstrated. Here, proteomics and CRISPR–Cas9 ablation of genes for specific proteins of the distal appendages of centrioles were used to map the hierarchy of distal appendage protein assembly that converts a daughter centriole into a mother centriole for ciliogenesis. The assembly map enables a molecular basis to understand how cells distinguish daughter from mother centrioles with cilia formation from only the latter.

Flagella of mammalian sperm have also been studied with an integrative approach, combining information from cryo-electron tomography, quantitative proteomics, and AI with AlphaFold2 for atomic resolution of proteins. The authors indicate this integrative approach as the virtual microscope (45, 46). It remains the proteomics of flagella based on prior work that is the foundation of the virtual microscope, for example (47, 48, 49). In this way, atomic models of motile cilia have been resolved (50).

**Fig. 7:**
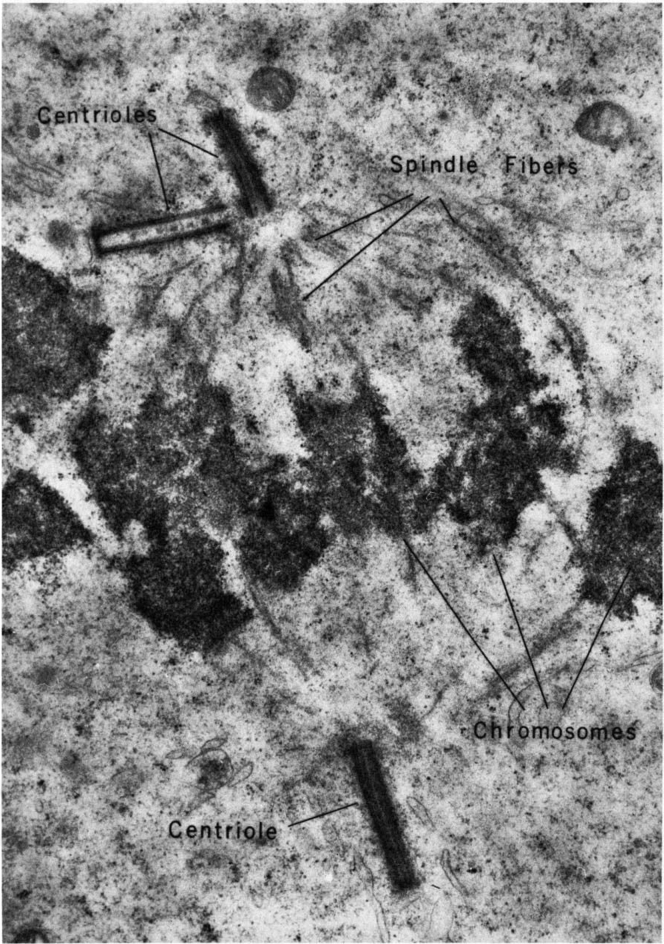
**Centrioles and spindle in a dividing spermatocyte showing the paired centrioles, spindle fibers, and chromosomes although microtubules are not preserved.** Reproduced from Fawcett 1981 (Fig. 302) (2).

The work has also extended the cryo-electron tomography and proteomics studies of the complete proteome of nonciliated thymus centrosomes (51). Here as in several types of dividing cells but not all, the centrosome organizes the mitotic spindle as visualized by pioneers in cell biology (Fig. 7). The paired centrioles assure accurate chromosome segregation during mitosis as well as the delivery of centrioles to new cells after mitosis (52). The association of centrosome microtubules with chromosomes is *via* the kinetochore on centromeric DNA of paired chromosomes.

## Mitotic Chromosomes

**Fig. 8:**
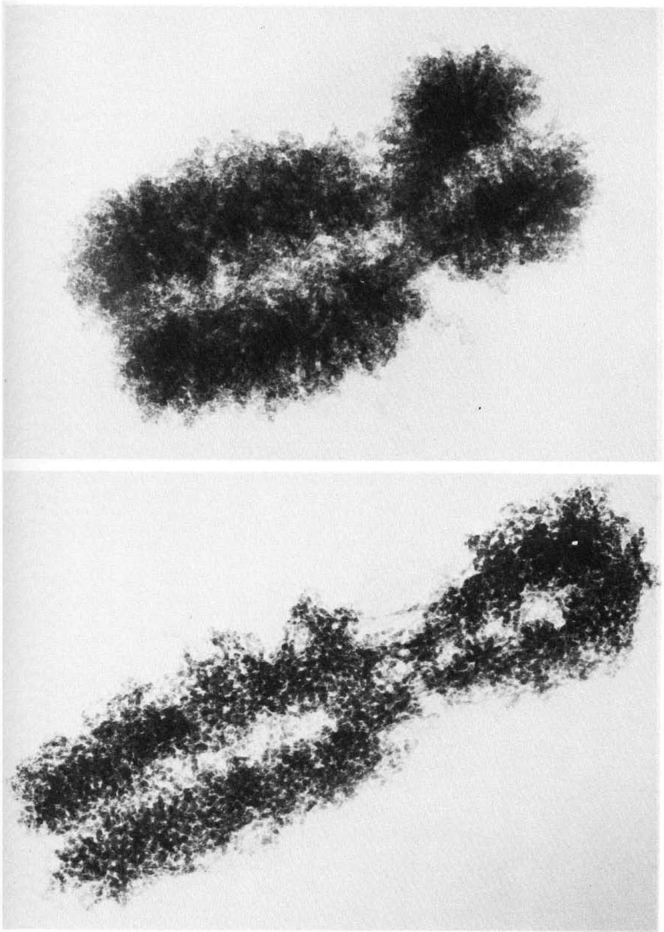
**High-voltage EM of CHO cell metaphase chromosome prepared in the absence of calcium.***Lower*, CHO metaphase chromosome isolated in the presence of calcium. Reproduced from Fawcett 1981 (Figs. 128 and 129) (2). CHO, Chinese hamster ovary cell line.

Mitotic microtubule-associated proteins were characterized by proteomics in 2001. Following the cell-free polymerization of microtubules in mitotic extracts of HeLa cells, and separation through discontinuous sucrose gradient centrifugation, protein separation by SDS-PAGE, and tandem mass spectrometry of tryptic peptides enabled protein characterization. Of the 15 proteins uncovered, astrin, a previously unreported coiled-coil protein, was found to localize to kinetochores with chromosomes aligned at the metaphase plate (53). In 2005, human metaphase chromosomes (shown here by EM from past cell biology pioneers, Fig. 8) were isolated from synchronized cells, with protein separation by SDS-PAGE or 2D gels with bands excised, digested, and tandem MS by MALDI TOF/TOF mass spectrometry with 158 proteins characterized including eight subunits of condensin I and II complexes (54).

In 2010, a methodology of protein multiclassifier combinatorial proteomics revealed 4000 proteins in isolated mitotic chromosomes (55). Random Forest–based machine learning then integrated the classifiers with a separate bioinformatics classifier. The authors tested 50 previously uncharacterized proteins found in mitotic chromosomes with 34 predicted by the multiclassifier to be on mitotic chromosomes with 30 confirmed by GFP tagging expression and microscopy. All known centromeric subcomplexes were thus identified but also an additional 110 not previously known as kinetochore associated. The multiclassifier strategy for proteomics has approached a comprehensive, accurate, and permanent characterization of proteins of mitotic chromosomes.

A further extension of the strategy was the application to the SMC, the structural maintenance of chromosome protein complexes. As before, mitotic chromosomes were isolated from nocodazole-treated DT40 cells. Stable isotope labeling by amino acids in cell culture comparisons of proteins were characterized by tandem mass spectrometry of wildtype and cells with conditional KO of SMC2, CAP-H, CAP-D3, or SMC5 to test for the dependence on members of the condensin, cohesin, and SM5 complex on proteins associated with mitotic chromosomes. Here, nano Random Forest machine learning based on the previous multiclassifier with combinatorial proteomics was used to integrate the proteomics datasets (56). The nano Random Forest strategy concluded that 113 of the 5038 proteins characterized were required for chromosome structure and segregation with one third of previously known. Proteins linked to kinetochore function were tested by siRNA and GFP-tagged proteins expressed and visualized in mitosis on mitotic chromosomes in cells. The study established the proteins functionally associated with mitotic chromosomes regulated by condensin I, condensin II, cohesion, SMC5/6, and Scc2/4 as well as their interdependencies (57). Proteomics has established a comprehensive, accurate, and permanent dataset of proteins needed for chromosome shape in mitosis. These are regulated by condensin I, condensin II complexes, the cohesin complex, chromokinesin KIF4A, and topoisomerase II alpha (58).

Protein structures from cryo-EM are now giving insight into kinetochore architecture (59). For the structural maintenance of chromosomal protein complexes, condensin, cohesin, and SMC5, 3D protein structures have revealed a common 40 nm diameter ring with coiled coil SMC proteins with insight into the mechanism of DNA loop extrusion (reviewed in Ref. (60)). It is remarkable, that the AI of AlphaFold to compare cohesin subcomplexes from different species enabled an understanding of mutations not previously interpretable (61). Also, insight into ternary complexes and the quaternary complex of Wapl, Pds5, SA/Scc3, and Scc1 were predicted. The AI-based structures have given impetus to study previously unknown interactions between cohesin subunits, how cohesin extrudes DNA loops, how cohesin’s exit and entry gates are regulated, and cohesin dissociation from chromosomes with several predictions for mechanisms that are under test experimentally (61, 62).

## The Ribosome

The aforementioned sample is of selected discoveries that have attained CAP or a near CAP compendium of proteins to resolve cell structure and mechanistic function. An earlier effort was achieved for the ribosome. First visualized by EM by Palade in 1955 (63), proteins were later characterized after ribosome isolation, protein separation and purification from 2D gels, and protein crosslinking and immuno-EM for bacterial ribosomes (64). Functional reconstitution followed for bacterial ribosomes and extension to the characterization of proteins in ribosomes from budding yeast as a model eukaryote (65). Further advances continued using proteomics (*e.g.*, (66, 67)), and integration with cryo-EM (68). The complete solution of ribosome structure was attained with mechanistic insight into function (*e.g.*, (69, 70, 71, 72)). The proteomics of ribosomes continues to generate discoveries (*e.g.*, (73)) as does imaging by time-resolved cryo-EM (74). For the latter, cryo-EM has resolved GTP-dependent structural intermediates with a subnanometer spatial resolution and a time resolution of milliseconds.

## Other Structures and Organelles

Protein characterization of cell structures including organelles has continued to advance remarkably as indicated in several reviews (75, 76, 77, 78, 79, 80, 81, 82, 83, 84, 85, 86, 87, 88, 89, 90). Indeed, organelle proteomics has a long history of success (*e.g.*, (81, 82, 83, 84)), awaiting integration. The isolation approach is complemented and extended for cell structures using proximity labeling including BioID (91). As proteomics continues unabated, the integration of the datasets with cryotomography (23) and AI (92) for not only cell structures but domains such as those of the endoplasmic reticulum for interaction complexes with mitochondria, endosomes, and other organelles (93) will be forthcoming. Experimental verification of the AI predictions, however, may be necessary to ensure the CAP principle is achieved (94).

It is the eventual application of the molecular/virtual Microscope as indicated in the graphical abstract that will resolve the molecular anatomy and functional mechanism of each structure, organelle, compartment of the cell, and all interaction domains between and among cell structures as based on the foundation of proteomics. For Cell Biology, the progress from EM descriptions of cell structures (2) to the proteomics-based molecular microscope has been noteworthy. Such advances with a CAP-assured characterization of proteins to integrate with imaging will resolve all cell structures and provide mechanistic insight into function (95, 96).

## Conflict of interest

The author declares no competing interests.

## References

[1] Klykov O., Kopylov M., Carragher B., Heck A.J.R., Noble A.J., Scheltema R.A. (2022). Label-free visual proteomics: coupling MS- and EM-based approaches in structural biology. Mol. Cell.

[2] Fawcett D.W. (1981).

[3] Crombie C., Junio A., Fraser A. (2003). The not-so-humble worm. Genome Biol..

[4] White J.G., Southgate E., Thomson J.N., Brenner S. (1986). The structure of the nervous system of the nematode caenorhabditis elegans. Philos. Trans. R. Soc. Lond. B Biol. Sci..

[5] White J. (2020). Of worms and men. J. Neurogenet..

[6] Ahmad Y., Lamond A.I. (2014). A perspective on proteomics in cell biology. Trends Cell Biol..

[7] Lundberg E., Borner G.H.H. (2019). Spatial proteomics: a powerful discovery tool for cell biology. Nat. Rev. Mol. Cell Biol..

[8] Rout M.P., Blobel G. (1993). Isolation of the yeast nuclear pore complex. J. Cell Biol..

[9] Chait B.T., Cadene M., Olinares P.D., Rout M.P., Shi Y. (2016). Revealing higher order protein structure using mass spectrometry. J. Am. Soc. Mass Spectrom..

[10] Rout M.P., Aitchison J.D., Suprapto A., Hjertaas K., Zhao Y., Chait B.T. (2000). The yeast nuclear pore complex: composition, architecture, and transport mechanism. J. Cell Biol..

[11] Wente S.R., Rout M.P. (2010). The nuclear pore complex and nuclear transport. Cold Spring Harb. Perspect. Biol..

[12] Alber F., Dokudovskaya S., Veenhoff L.M., Zhang W., Kipper J., Devos D. (2007). The molecular architecture of the nuclear pore complex. Nature.

[13] Ori A., Banterle N., Iskar M., Andres-Pons A., Escher C., Khanh Bui H. (2013). Cell type-specific nuclear pores: a case in point for context-dependent stoichiometry of molecular machines. Mol. Syst. Biol..

[14] Ori A., Andres-Pons A., Beck M. (2014). The use of targeted proteomics to determine the stoichiometry of large macromolecular assemblies. Methods Cell Biol..

[15] LaCava J., Fernandez-Martinez J., Hakhverdyan Z., Rout M.P. (2016). Optimized affinity capture of yeast protein complexes. Cold Spring Harb Protoc..

[16] LaCava J., Jiang H., Rout M.P. (2016). Protein complex affinity capture from Cryomilled mammalian cells. J. Vis. Exp..

[17] LaCava J., Fernandez-Martinez J., Hakhverdyan Z., Rout M.P. (2016). Protein complex purification by affinity capture. Cold Spring Harb. Protoc..

[18] Subbotin R.I., Chait B.T. (2014). A pipeline for determining protein-protein interactions and proximities in the cellular milieu. Mol. Cell. Proteomics.

[19] Hakhverdyan Z., Molloy K.R., Subbotin R.I., Fernandez-Martinez J., Chait B.T., Rout M.P. (2021). Measuring *in vivo* protein turnover and exchange in yeast macromolecular assemblies. STAR Protoc..

[20] Kim S.J., Fernandez-Martinez J., Nudelman I., Shi Y., Zhang W., Raveh B. (2018). Integrative structure and functional anatomy of a nuclear pore complex. Nature.

[21] Kim D.I., Birendra K.C., Zhu W., Motamedchaboki K., Doye V., Roux K.J. (2014). Probing nuclear pore complex architecture with proximity-dependent biotinylation. Proc. Natl. Acad. Sci. U. S. A..

[22] Bui K.H., von Appen A., DiGuilio A.L., Ori A., Sparks L., Mackmull M.T. (2013). Integrated structural analysis of the human nuclear pore complex scaffold. Cell.

[23] Ochner H., Bharat T.A.M. (2023). Charting the molecular landscape of the cell. Structure.

[24] Akey C.W., Singh D., Ouch C., Echeverria I., Nudelman I., Varberg J.M. (2022). Comprehensive structure and functional adaptations of the yeast nuclear pore complex. Cell.

[25] Akey C.W., Echeverria I., Ouch C., Nudelman I., Shi Y., Wang J. (2023). Implications of a multiscale structure of the yeast nuclear pore complex. Mol. Cell.

[26] Cowburn D., Rout M. (2023). Improving the hole picture: towards a consensus on the mechanism of nuclear transport. Biochem. Soc. Trans..

[27] Yu M., Heidari M., Mikhaleva S., Tan P.S., Mingu S., Ruan H. (2023). Visualizing the disordered nuclear transport machinery *in situ*. Nature.

[28] Mackmull M.T., Klaus B., Heinze I., Chokkalingam M., Beyer A., Russell R.B. (2017). Landscape of nuclear transport receptor cargo specificity. Mol. Syst. Biol..

[29] Cronshaw J.M., Krutchinsky A.N., Zhang W., Chait B.T., Matunis M.J. (2002). Proteomic analysis of the mammalian nuclear pore complex. J. Cell Biol..

[30] Leitner A., Walzthoeni T., Kahraman A., Herzog F., Rinner O., Beck M. (2010). Probing native protein structures by chemical cross-linking, mass spectrometry, and bioinformatics. Mol. Cell. Proteomics.

[31] von Appen A., Kosinski J., Sparks L., Ori A., DiGuilio A.L., Vollmer B. (2015). *In situ* structural analysis of the human nuclear pore complex. Nature.

[32] Mosalaganti S., Obarska-Kosinska A., Siggel M., Taniguchi R., Turonova B., Zimmerli C.E. (2022). AI-based structure prediction empowers integrative structural analysis of human nuclear pores. Science.

[33] Zimmerli C.E., Allegretti M., Rantos V., Goetz S.K., Obarska-Kosinska A., Zagoriy I. (2021). Nuclear pores dilate and constrict in cellulo. Science.

[34] Zhao L., Hou Y., Picariello T., Craige B., Witman G.B. (2019). Proteome of the central apparatus of a ciliary axoneme. J. Cell Biol..

[35] Han L., Rao Q., Yang R., Wang Y., Chai P., Xiong Y. (2022). Cryo-EM structure of an active central apparatus. Nat. Struct. Mol. Biol..

[36] Kubo S., Black C.S., Joachimiak E., Yang S.K., Legal T., Peri K. (2023). Native doublet microtubules from Tetrahymena thermophila reveal the importance of outer junction proteins. Nat. Commun..

[37] Ghanaeian A., Majhi S., McCafferty C.L., Nami B., Black C.S., Yang S.K. (2023). Integrated modeling of the Nexin-dynein regulatory complex reveals its regulatory mechanism. Nat. Commun..

[38] Walton T., Gui M., Velkova S., Fassad M.R., Hirst R.A., Haarman E. (2023). Axonemal structures reveal mechanoregulatory and disease mechanisms. Nature.

[39] Legal T., Parra M., Tong M., Black C.S., Joachimiak E., Valente-Paterno M. (2023). CEP104/FAP256 and associated cap complex maintain stability of the ciliary tip. J. Cell Biol..

[40] Ichikawa M., Khalifa A.A.Z., Kubo S., Dai D., Basu K., Maghrebi M.A.F. (2019). Tubulin lattice in cilia is in a stressed form regulated by microtubule inner proteins. Proc. Natl. Acad. Sci. U. S. A..

[41] Bettencourt-Dias M., Hildebrandt F., Pellman D., Woods G., Godinho S.A. (2011). Centrosomes and cilia in human disease. Trends Genet..

[42] Kumar D., Reiter J. (2021). How the centriole builds its cilium: of mothers, daughters, and the acquisition of appendages. Curr. Opin. Struct. Biol..

[43] Carden S., Vitiello E., Rosa E.S.I., Holder J., Quarantotti V., Kishore K. (2023). Proteomic profiling of centrosomes across multiple mammalian cell and tissue types by an affinity capture method. Dev. Cell.

[44] Roux K.J., Kim D.I., Raida M., Burke B. (2012). A promiscuous biotin ligase fusion protein identifies proximal and interacting proteins in mammalian cells. J. Cell Biol..

[45] Chen Z., Shiozaki M., Haas K.M., Skinner W.M., Zhao S., Guo C. (2023). De novo protein identification in mammalian sperm using *in situ* cryoelectron tomography and AlphaFold2 docking. Cell.

[46] Chen Z., Greenan G.A., Shiozaki M., Liu Y., Skinner W.M., Zhao X. (2023). *In situ* cryo-electron tomography reveals the asymmetric architecture of mammalian sperm axonemes. Nat. Struct. Mol. Biol..

[47] Linck R., Fu X., Lin J., Ouch C., Schefter A., Steffen W. (2014). Insights into the structure and function of ciliary and flagellar doublet microtubules: tektins, Ca2+-binding proteins, and stable protofilaments. J. Biol. Chem..

[48] Baker M.A., Hetherington L., Reeves G.M., Aitken R.J. (2008). The mouse sperm proteome characterized via IPG strip prefractionation and LC-MS/MS identification. Proteomics.

[49] Firat-Karalar E.N., Sante J., Elliott S., Stearns T. (2014). Proteomic analysis of mammalian sperm cells identifies new components of the centrosome. J. Cell Sci..

[50] Grossman-Haham I. (2023). Towards an atomic model of a beating ciliary axoneme. Curr. Opin. Struct. Biol..

[51] Busselez J., Chichon F.J., Rodriguez M.J., Alpizar A., Gharbi S.I., Franch M. (2019). Cryo-Electron Tomography and Proteomics studies of centrosomes from differentiated quiescent thymocytes. Sci. Rep..

[52] Hoffmann I. (2021). Centrosomes in mitotic spindle assembly and orientation. Curr. Opin. Struct. Biol..

[53] Mack G.J., Compton D.A. (2001). Analysis of mitotic microtubule-associated proteins using mass spectrometry identifies astrin, a spindle-associated protein. Proc. Natl. Acad. Sci. U. S. A..

[54] Uchiyama S., Kobayashi S., Takata H., Ishihara T., Hori N., Higashi T. (2005). Proteome analysis of human metaphase chromosomes. J. Biol. Chem..

[55] Ohta S., Bukowski-Wills J.C., Sanchez-Pulido L., Alves Fde L., Wood L., Chen Z.A. (2010). The protein composition of mitotic chromosomes determined using multiclassifier combinatorial proteomics. Cell.

[56] Montano-Gutierrez L.F., Ohta S., Kustatscher G., Earnshaw W.C., Rappsilber J. (2017). Nano Random Forests to mine protein complexes and their relationships in quantitative proteomics data. Mol. Biol. Cell.

[57] Ohta S., Montano-Gutierrez L.F., de Lima Alves F., Ogawa H., Toramoto I., Sato N. (2016). Proteomics analysis with a nano random forest approach reveals novel functional interactions regulated by SMC complexes on mitotic chromosomes. Mol. Cell. Proteomics.

[58] Paulson J.R., Hudson D.F., Cisneros-Soberanis F., Earnshaw W.C. (2021). Mitotic chromosomes. Semin. Cell Dev. Biol..

[59] Ariyoshi M., Fukagawa T. (2023). An updated view of the kinetochore architecture. Trends Genet..

[60] Dekker C., Haering C.H., Peters J.M., Rowland B.D. (2023). How do molecular motors fold the genome?. Science.

[61] Nasmyth K.A., Lee B.G., Roig M.B., Lowe J. (2023). What AlphaFold tells us about cohesin's retention on and release from chromosomes. Elife.

[62] Ochs F., Green C., Szczurek A.T., Pytowski L., Kolesnikova S., Brown J. (2024). Sister chromatid cohesion is mediated by individual cohesin complexes. Science.

[63] Palade G.E. (1955). A small particulate component of the cytoplasm. J. Biophys. Biochem. Cytol..

[64] Brimacombe R., Stoffler G., Wittmann H.G. (1978). Ribosome structure. Annu. Rev. Biochem..

[65] Nomura M. (1999). Regulation of ribosome biosynthesis in Escherichia coli and Saccharomyces cerevisiae: diversity and common principles. J. Bacteriol..

[66] Benjamin D.R., Robinson C.V., Hendrick J.P., Hartl F.U., Dobson C.M. (1998). Mass spectrometry of ribosomes and ribosomal subunits. Proc. Natl. Acad. Sci. U. S. A..

[67] Gordiyenko Y., Videler H., Zhou M., McKay A.R., Fucini P., Biegel E. (2010). Mass spectrometry defines the stoichiometry of ribosomal stalk complexes across the phylogenetic tree. Mol. Cell. Proteomics.

[68] Gilbert R.J., Fucini P., Connell S., Fuller S.D., Nierhaus K.H., Robinson C.V. (2004). Three-dimensional structures of translating ribosomes by Cryo-EM. Mol. Cell.

[69] Ogle J.M., Ramakrishnan V. (2005). Structural insights into translational fidelity. Annu. Rev. Biochem..

[70] Ben-Shem A., Garreau de Loubresse N., Melnikov S., Jenner L., Yusupova G., Yusupov M. (2011). The structure of the eukaryotic ribosome at 3.0 A resolution. Science.

[71] Ramakrishnan V. (2014). The ribosome emerges from a black box. Cell.

[72] Khatter H., Myasnikov A.G., Natchiar S.K., Klaholz B.P. (2015). Structure of the human 80S ribosome. Nature.

[73] Lai S.H., Tamara S., Heck A.J.R. (2021). Single-particle mass analysis of intact ribosomes by mass photometry and Orbitrap-based charge detection mass spectrometry. iScience.

[74] Bhattacharjee S., Feng X., Maji S., Dadhwal P., Zhang Z., Brown Z.P. (2024). Time resolution in cryo-EM using a PDMS-based microfluidic chip assembly and its application to the study of HflX-mediated ribosome recycling. Cell.

[75] Christopher J.A., Geladaki A., Dawson C.S., Vennard O.L., Lilley K.S. (2022). Subcellular Transcriptomics and proteomics: a comparative methods review. Mol. Cell. Proteomics.

[76] Hevler J.F., Heck A.J.R. (2023). Higher-order structural Organization of the mitochondrial proteome Charted by in situ cross-linking mass spectrometry. Mol. Cell. Proteomics.

[77] Naba A. (2023). Ten years of extracellular matrix proteomics: accomplishments, challenges, and future perspectives. Mol. Cell. Proteomics.

[78] Borner G.H.H. (2020). Organellar maps through proteomic profiling - a conceptual Guide. Mol. Cell. Proteomics.

[79] Samavarchi-Tehrani P., Samson R., Gingras A.C. (2020). Proximity dependent biotinylation: key enzymes and adaptation to proteomics approaches. Mol. Cell. Proteomics.

[80] Rees J.S., Li X.W., Perrett S., Lilley K.S., Jackson A.P. (2015). Protein neighbors and proximity proteomics. Mol. Cell. Proteomics.

[81] Andersen J.S., Mann M. (2006). Organellar proteomics: turning inventories into insights. EMBO Rep..

[82] Brunet S., Thibault P., Gagnon E., Kearney P., Bergeron J.J., Desjardins M. (2003). Organelle proteomics: looking at less to see more. Trends Cell Biol..

[83] Pflieger D., Rossier J. (2008). Organelle proteomics. Preface. Methods Mol. Biol..

[84] Udi Y., Zhang W., Stein M.E., Ricardo-Lax I., Pasolli H.A., Chait B.T. (2023). A general method for quantitative fractionation of mammalian cells. J. Cell Biol..

[85] Au C.E., Bell A.W., Gilchrist A., Hiding J., Nilsson T., Bergeron J.J. (2007). Organellar proteomics to create the cell map. Curr. Opin. Cell Biol..

[86] Hermo L., Oliveira R.L., Smith C.E., Au C.E., Bergeron J.J.M. (2018).

[87] Yates J.R., Gilchrist A., Howell K.E., Bergeron J.J. (2005). Proteomics of organelles and large cellular structures. Nat. Rev. Mol. Cell Biol..

[88] Smirle J., Au C.E., Jain M., Dejgaard K., Nilsson T., Bergeron J. (2013). Cell biology of the endoplasmic reticulum and the Golgi apparatus through proteomics. Cold Spring Harb. Perspect. Biol..

[89] Gannon J., Bergeron J.J., Nilsson T. (2011). Golgi and related vesicle proteomics: simplify to identify. Cold Spring Harb. Perspect. Biol..

[90] Reghupaty S.C., Dall N.R., Svensson K.J. (2023). Hallmarks of the metabolic secretome. Trends Endocrinol. Metab..

[91] Milione R.R., Schell B.B., Douglas C.J., Seath C.P. (2023). Creative approaches using proximity labeling to gain new biological insights. Trends Biochem. Sci..

[92] Bordin N., Lau A.M., Orengo C. (2023). Large-scale clustering of AlphaFold2 3D models shines light on the structure and function of proteins. Mol. Cell.

[93] Phillips M.J., Voeltz G.K. (2016). Structure and function of ER membrane contact sites with other organelles. Nat. Rev. Mol. Cell Biol..

[94] Terwilliger T.C., Liebschner D., Croll T.I., Williams C.J., McCoy A.J., Poon B.K. (2023). AlphaFold predictions are valuable hypotheses and accelerate but do not replace experimental structure determination. Nat. Methods.

[95] Beck M., Covino R., Hanelt I., Muller-McNicoll M. (2024). Understanding the cell: future views of structural biology. Cell.

[96] McCafferty C.L., Klumpe S., Amaro R.E., Kukulski W., Collinson L., Engel B.D. (2024). Integrating cellular electron microscopy with multimodal data to explore biology across space and time. Cell.

